# Enhancing antioxidant activity and quality of *Triadica cochinchinensis* honey via an automated temperature-humidity controlled cabinet

**DOI:** 10.3389/fnut.2025.1641551

**Published:** 2025-09-24

**Authors:** Huizhi Jiang, Weixuan Chen, Wujun Jiang, Feng Liu, Xiaobo Wu, Weiyu Yan, Xujiang He, Zhijiang Zeng

**Affiliations:** ^1^Honeybee Research Institute, Jiangxi Agricultural University, Nanchang, China; ^2^Jiangxi Province Key Laboratory of Honeybee Biology and Beekeeping, Nanchang, China; ^3^Apiculture Research Institute of Jiangxi Province, Nanchang, China

**Keywords:** honey, honey cabinet, chemical profiling, bioactivity, antioxidant activity

## Abstract

Honey, a key beekeeping product, is rich in antioxidants and bioactive compounds, offering antimicrobial, anti-inflammatory and health-promoting properties. The water content of honey is directly correlated with its quality. However, *Triadica cochinchinensis* honey (TCH), produced in high humidity regions, is frequently at risk of fermentation and spoilage due to excessive water content. A dewatering method using an automated, temperature- and humidity-controlled honey cabinet was applied to address this issue and investigate its effects on TCH. After 96 h of treatment, the water content of TCH capped honeycombs effectively reduced to below 18%. Meanwhile, most physicochemical parameters, volatile compounds and chemical compositions largely remained unchanged, thereby preserving their nutritional value and flavor. Moreover, phenolics and flavonoids levels significantly increased by 15.83 and 25.42%, respectively, thereby enhancing the honey’s antioxidant capacity. Our results indicated that this method can significantly enhance maturity and improve the quality of TCH, providing reference value for other honey produced in high humidity regions. The utilization of honey cabinets can enhance the market value of honey in these regions, and consumers can benefit from higher-quality honey with greater biological activity.

## 1 Introduction

Honey, a sweet food, is collected by worker bees from the nectar or plant secretions, which is subsequently processed and stored in honeycombs (1, 2). It is a rich source of natural antioxidants and exhibits various beneficial properties, including antioxidant (3) antibacterial (4), and anti-inflammatory effects (5). Composed primarily of glucose and fructose, honey contains sugars that are easily absorbed by the human body (6). In addition, honey is packed with enzymes, vitamins, trace elements, and organic acids, contributing to its significant nutritional value (7).

Antioxidant capacity is one of the most important properties of honey (8). It depends on concentration, botanical source, geographical origin, processing methods and storage conditions (9, 10). Phenolic compounds, mainly responsible for honey’s antioxidant properties, are secondary metabolites produced by plants and transferred to honey through bee foraging (11, 12). The phenolic compounds in honey mainly consist of flavonoids and phenolic acids (13). Additionally, amino acids, antioxidant enzymes, and vitamins in honey also contribute to its antioxidant capacity (14).

The water content of honey is a key factor in determining its physicochemical properties, affecting its quality, storage stability, crystallization, and viscosity (15). Several factors influence honey’s water content, including the type of nectar source, bee species, colony conditions, honey production duration, and environmental temperature and humidity during the nectar flow period, as well as storage methods (16). For instance, in regions with high humidity, such as Southern China and tropical countries, the water content of capped honey can range from 22 to 23% (17, 18). If honey is extracted directly without proper processing, the excessive water content may lead to fermentation, spoilage, flavor degradation, phase separation, and ultimately compromise both the quality and safety of honey for consumption (19, 20). The standard in China specifies a maximum water content of 18% for mature honey and 20% for regular honey. The lower water content effectively inhibits yeast growth and extends storage life (21, 22).

**Figure 1:**
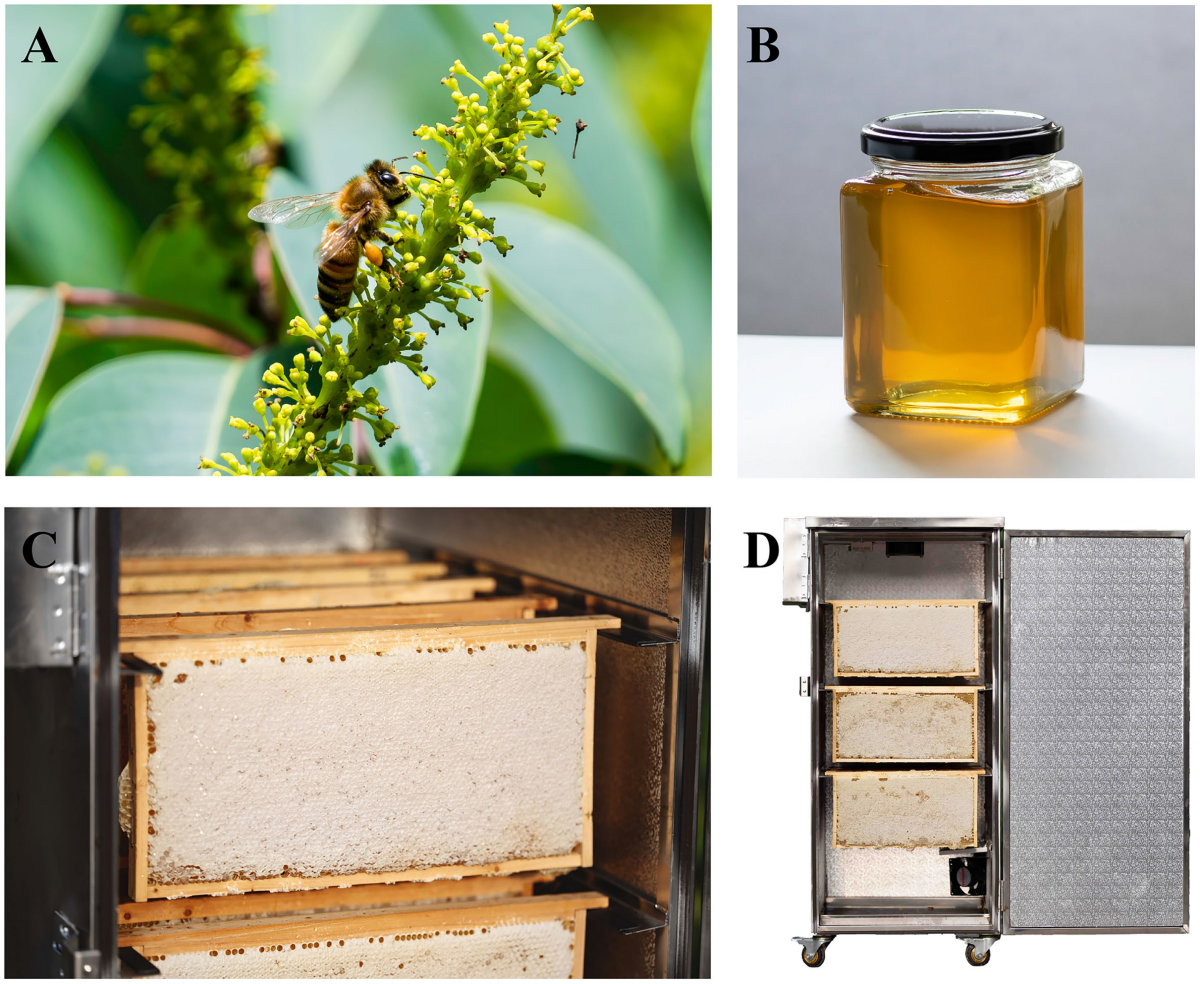
The botanical original analysis of *Triadica cochinchinensis* monofloral honey (TCH) and running operation of honey cabinet. **(A)** Honeybee visited *T. cochinchinensis* flower. **(B)** View of TCH. **(C)** The capped honeycombs of TCH in the honey cabinet. **(D)** Physical map of honey cabinet. Dimensions: 1.2 m (W) × 0.8 m (D) × 1.6 m (H). Capacity: 15 honeycombs (40 × 25 cm each).

*Triadica cochinchinensis* is one of the primary nectar sources in southern China, with its flowering period occurring from June to July and characterized by large nectar secretion (Figure 1A) (23). *Triadica cochinchinensis* honey (TCH) has the highest production in southern China during summer, and each colony (*Apis mellifera*) can produce 20 to 30 kilograms of TCH (Figure 1B) (24). Previous studies have shown that TCH is notably rich in iron and zinc, exhibits naturally low diastase activity and possesses the ability to alleviate alcoholic liver damage and anti-aging properties (25–27). (−)-Gallocatechin gallate is a characteristic marker to identify TCH, which belongs to flavonoids and exhibits antioxidant properties (18). The southern regions of China have typical subtropical monsoon climate, featuring hot and rainy summers (28). Due to the high humidity levels in the production environment, TCH tends to have a relatively high water content (> 22%) after being capped, and most of it is immature. This not only makes it easy to ferment and spoilage but also leads to coarse crystallization—a defect in which sugars form large, gritty crystals. Crystallization compromises its texture and consumer acceptability and ultimately resulted in a low selling price for TCH and reduced income for beekeepers (29, 30). Over time, there has been a huge negative impact on the regional economy because of the difficulty in marketing low quality TCH.

Several methods are employed to address the challenge of high water content in honey production. Thermal treatment is commonly employed for industrial honey dehydration, with temperature typically ranging from 55 °C to 80 °C (31). However, high-temperatures may reduce diastase activity and induce the formation of harmful substances such as 5-hydroxymethylfurfural (HMF) (32, 33). As a potential carcinogen, HMF is correlated with reduced honey freshness (34). Thermal treatment can enhance honey’s antioxidant activity (35). Vacuum drying methods of honey include high-temperature, freeze and ultrasonic vacuum drying. High-temperature and freeze vacuum drying can increase the total phenolics and flavonoid level of honey (15), whereas ultrasonic vacuum drying increases HMF content and darkens honey color (36). Microwave heating is another method for honey drying, as it penetrates and interacts with honey to achieve rapid heating (37). Nevertheless, under the same processing time, HMF formation seems to increase to a greater extent compared to thermal heating, and there is a decrease in glucose oxidase and invertase activity (38). When stingless bee honey is subjected to microwave heating at a constant power level of 60 W for 1 min, its antioxidant activity significantly decreases (39). Placing capped honeycombs in a hot room equipped with an automated temperature (36–38 °C) and humidity (30–40%) control system is also a useful drying method. This approach replicates the temperature and humidity conditions within bee colonies to facilitate honey dehydration. After 3 to 4 days of treatment, this method can effectively reduce the water content of the honey to below 18% (40–42).

For small-scale apiaries (<100 colonies), thermal treatment may reduce the nutritional value of honey. Vacuum drying and microwave heating require skilled operators and are difficult to maintain. Hot rooms are impractical due to spatial limitations (>10 m^2^) and financial constraints. However, beekeepers in small apiaries are in urgent need of a low-cost and quality-preserving way to reduce the water of honey.

This study, drawing on the principles of hot air-drying technology, developed an automated temperature- and humidity-controlled honey cabinet designed explicitly for small apiaries in high-humidity regions to reduce the water content of TCH. The cabinet (1.5 m^3^) utilizes heaters, dehumidifiers, and axial fans to maintain a temperature of 38 °C and a relative humidity of 30% (Supplementary Figures S1, S2), processing 15 honeycombs per batch. Comprehensive functional verification demonstrated that after treatment with the cabinet, the water content in capped honeycombs of TCH was reduced to below 18%, while effectively preserving nutritional and flavor components of honey and enhancing its antioxidant capacity. Unlike industrial dehydrators or hot rooms, its compact design (<0.5 kW in power) suited small apiaries, offering the advantages of low operating costs, quick return on investment and portability. This innovation not only enhances honey quality and concentration but also contributes to improved economic returns for beekeepers.

## 2 Materials and methods

### 2.1 Sample collection

TCH was selected for this experiment, which had the largest production during summer in the high-humidity regions of southern China. The apiaries selected for the production and harvesting of TCH were Weimin apiary in Yongxiu County, Jiangxi Province, China (29°01′N, 115°29′E) and Gan’s apiary in Anfu County, Jiangxi Province, China (114°21′E, 27°14’N). Both were surrounded by plantations of *T. cochinchinensis*. During the flowering period of *T. cochinchinensis* (June–July 2024), 10 colonies (*Apis mellifera*) were organized to produce TCH. Honeycombs with a capped rate exceeding 95% were chosen as the test samples.

### 2.2 Chemicals and reagents

LC–MS acetonitrile, methanol and formic acid were purchased from Thermo Fisher (MA, United States). A mixture of n-alkanes (C7-C40) standard was purchased from Merck (Darmstadt, Germany). Glucose and fructose standards were obtained from Dr. Ehrenstorfer (Augsburg, Germany). Sucrose standard was sourced from Anpel (Shanghai, China). Octanol standard was purchased from TMTR (Changzhou, China). Rutin and gallic acid standards from Weiye (Beijing, China). Furfural standard solution was acquired from Alta (Tianjin, China). NaOH and H_2_SO_4_ were purchased from Bolind (Shenzhen, China). Glycerol, sodium acetate, acetic acid, iodine, potassium iodide, sodium chloride, and starch were purchased from Sinopharm (Shanghai, China). Dichloromethane (purity) ≥ 99.9% was purchased from Xiya (Linyi, China). AlCl_3_, Na_2_CO_3_ and ethanol were obtained from Xilong (Shantou, China). Honey glycerol enzymatic analysis kit was obtained from Kwinbon (Beijing, China). Folin-Ciocâlteu reagent, ABTS free radical scavenging capacity (ABTS+) and ferricion reducing antioxidant power (FRAP) kits were purchased from yuanye (Shanghai, China). 2,2-diphenyl-1-picrylhydrazyl (DPPH) was acquired from Phygene (Fujian, China). Ultra-pure water from Ultrapure Lab Water Systems (RephiLe, Shanghai, China) was used throughout the study.

### 2.3 Honey cabinet and verification of its dewatering efficiency and energy consumption

A honey cabinet was used to excessive water content in this experiment. The control system of the honey cabinet utilizes the temperature and humidity sensor to automatically regulate the heater, dehumidifier, and axial fan, ensuring proper air circulation and maintaining stable temperature and humidity environment within the honey cabinet (Supplementary Figures S1, S2). Dimensions: 1.2 m (W) × 0.8 m (D) × 1.6 m (H). Capacity: 15 honeycombs (40 × 25 cm each). The relevant parameters of honey cabinet were summarized in Supplementary Table S1. The automated control system can maintain a stable temperature at 38 °C ± 1 °C and the relative humidity at 30 ± 5%. Upon activation of the honey cabinet, the heated air is accelerated, distributed and filled the entire cavity, which creates a continuous circulation of hot air within the drying chamber and effectively facilitates the drying of the capped honeycombs (Supplementary Figures S3, S4). The operational flow diagram for the honey cabinet was shown in Supplementary Figure S5.

A total of 30 capped honeycombs of TCH (15 in one apiary) with an initial water content of 21–22% were selected. Each honeycomb was divided into six areas, and five cells were randomly sampled from each area to measure the water content, allowing for the estimation of the overall water content across the entire honeycomb. Five honeycombs were placed on each shelf of the honey cabinet, maintaining a spacing of 6–8 cm between them (Figures 1C,D).

We conducted a pre-experiment using capped honeycomb samples with different initial water contents and found that the water contents of all samples could be reduced to below 18% after 96 h of treatment. Therefore, the honeycombs were processed in the cabinet for 96 h. Before treatment (0 H), five honeycombs were randomly chosen. Each honeycomb was divided into six areas (three on each side), and five cells were selected within each section. Remove the beeswax caps and measure the initial water content of TCH. After 96 h of treatment (96 H), follow-up sampling was performed in each area by measuring water of five cells. Random sampling was performed in each honeycomb area, and the analysts were blinded to treatment groups during subsequent experiments.

There were 30 replicates in each group. The water content was then calculated by determining the refractive index at 40 °C using an Abbe refractometer (DRA1, ATAGO, Tokyo, Japan). During the cabinet run, the energy consumed by honey cabinet was recorded with power meters.

### 2.4 Palynological and physicochemical analysis

The TCH produced by Weimin apiary was utilized for subsequent analysis. The light microscope equipped with a camera (DS-Fi3, Nikon Corporation, Tokyo, Japan) was used to palynological analysis. The TCH samples preparation and local pollen grains determination was based on the method of Yang et al. (43).

To examine the effects of cabinet treatment on TCH quality, the physicochemical components of 0 and 96 H TCH samples were analyzed. The contents of fructose, glucose and sucrose in TCH samples were determined using the AOAC method No. 977.20 (44). A high-performance liquid chromatograph (HPLC) equipped with a refractive index detector (2695–2,414, Waters, MA, United States). The Cosmosil Sugar-D (5 μm 4.6 × 250 mm) column (Nacalai tesque, Kyoto, Japan) was used for the separation of sugar compounds. The mobile phase consisted of water-acetonitrile (25:75, v/v) with a flow rate at 1.0 mL/min, column temperature at 40 °C, injection volume of 20 μL and run time of 15 min per sample. Standard solutions of glucose, fructose and sucrose were prepared to establish standard curves for calculating the sample concentration. The water system also used for HMF analysis consisted of an HPLC and an ultraviolet (UV) detector. The chromatographic separation was by ACQUITY UPLC BEH C18 (1.7 μm 2.1 × 50 mm) column (Waters, MA, United States). The conditions were as follows: the mobile phase consisted of water–methanol (90:10, v/v), the flow rate of 1.0 mL/min, column temperature at 30 °C, injection volume of 10 μL and run time of 15 min per sample. HMF standards were prepared at concentrations of 0.01 mg/mL - 1 mg/mL with water–methanol (90:10, v/v) for calibration. Samples were dissolved to 100 mg/mL with water–methanol (90:10, v/v) for determination.

The diastase activity of TCH was analysis as follows: 5.0 g sample was dissolved in 15 mL water, together with 2.5 mL acetate buffer (pH = 5.3). Then 1.5 mL of 0.5 M sodium chloride was added, and the mixture was diluted to 25 mL with water. 5 mL starch solution was combined with 10 mL honey solution. From the 5th min, 1.0 mL of the mixture was added to 10.0 mL iodine solution every 15 min and then the absorbance was measured at 660 nm using a spectrophotometer. The procedure was repeated until the absorbance dropped below 0.235. A standard curve was drawn with time and absorbance to calculate the time when the absorbance of the sample mixed solution reached 0.235.

The free acidity was determined using the Chinese rules for the inspection of honey for import and export (SN/T0852-2012) (45). A 10.0 g honey sample was dissolved in 75 mL of boiled and cooled water. Two to three drops of 1% phenolphthalein indicator were added, followed by titration with 0.1 M sodium hydroxide solution until a stable pink color persisted for at least 10s. The final acidity values were calculated in mL/kg. The glycerol of TCH were assessed utilizing commercial assay kits and results were expressed as mg/kg.

The lab chromaticity was measured by a CM-5 chromameter (Minolta, Shanghai, China). The samples were filled into the cuvette and placed in the transmission sample chamber to measure the L* (lightness), a* (Redness) and b*(yellowness) values after whiteboard correction.

### 2.5 GC–MS analysis

The SPME fiber (50/30 μm DVB/CAR/PDMS, stableflex (2 cm) 24 Ga; Supelco, PA, United States) was used to extract volatile compounds from TCH samples. 5.000 g of sample was weighed, dissolved in distilled water, and diluted to 10 mL to obtain a concentration of 0.5 g/mL. The mixture was then transferred to a 20 mL SPME vial. 200 μL of octanol was subsequently added (dissolved in dichloromethane, 10 μg/mL) and mixed well (46). The samples were incubated at 40 °C for 15 min and then extracted for 30 min. Before the extraction, the SPME fibers were conditioned 30 min.

GC–MS system (5977B-7890B, Agilent Technologies, CA, United States) equipped with HP-VOC (30 m × 0.20 mm × 1.12 μm) was adopted for sample analysis. The conditions were as follows: Helium (99.99% purity) served as the carrier gas at a constant flow rate of 1.2 mL/min, maintaining a constant pressure of 9.1075 psi. The injection temperature was 250 °C and the analytes were carried out in splitless mode. The initial column temperature was held at 40 °C for 2 min, then increased to 180 °C at a rate of 2 °C/min, followed by a further increase to 260 °C at 5 °C/min, and held for 5 min. The total analysis time was 97.00 min. The mass spectra data were performed under 70 eV electron ionization.

### 2.6 UPLC-MS/MS analysis

The Oasis HLB (3 cc/60 mg, Waters Oasis, MLF, United States), a solid-phase extraction (SPE) column was used for the extraction of compounds from TCH samples. Briefly, 2.5 g honey sample was weighed, dissolved thoroughly in 5 mL of water, and then centrifuged at 8000 r/min for 10 min. The supernatant was retained. After the SPE column was activated according to the instructions, the supernatant was loaded onto the column for component enrichment. The column was then washed with water and thoroughly removed residual water. Subsequently, the column was eluted with 3 mL of methanol and dried under nitrogen gas. The eluate was redissolved in 1 mL of 80% methanol solution, filtered through a 0.22 μm filter membrane, and transferred to an injection vial for analysis. The quality control (QC) sample was prepared by mixing equal volumes of the injection solutions from all samples, which was used to monitor the stability of the analytical system.

An ultra-high-performance liquid chromatography system (Dionex Ultimate 3,000, Thermo Fisher Scientific, MA, United States) coupled to a Q Exactive mass spectrometer (MS, Thermo Fisher Scientific, MA, United States) with a heated electrospray ionization (HESI) source was used for testing the compounds of TCH samples. The separation was performed on an ZORBAX Eclipse Plus C18 chromatographic column (3.0 × 150 mm × 1.8 μm, Agilent Technologies, CA, United States) thermostated at 40 °C. A gradient elution program ran with mobile phase A (0.1% formic acid water) and B (methanol) as follows: 0–0.5 min, 95% A; 0.5–4 min, 95–40% A; 4–12 min, 40–5% A, 12–16 min, 5% A; and 16–20 min, 95% A. Each injection of 2 μL was loaded with a flow rate of 0.3 mL/min. All samples were collected in positive (ESI+) and negative ion (ESI-) switching full scan modes. The quality control (QC) sample was injected once for every five real samples to examine the stability of the entire detection process and was used for qualitatively determination scanned in the full scan/ddMS2 mode.

The HESI parameters were optimized as follows: sheath gas flow rate 40 L/min; aux. Gas flow rate 5 L/min; spray voltage 3,000 V for ESI- and 3,500 V for ESI+; capillary temperature 320 °C; S lens radio frequency voltage level of 60%; and aux. Gas heater temperature 350 °C. Full scan data (m/z 80–1,200) were acquired at a resolution of 70, 000 and ddMS2 was set at 17,500. The collision energies were 20, 30, and 40 eV. The automatic gain control (AGC) was set at 1 × 10^6^ and the maximum injection time was set to 50 ms. The scan rate was set at 1 scan/s. MS data was collected using Xcalibur software 4.0 (Thermo Fisher Scientific, MA, United States) and saved as Raw format files.

### 2.7 Analysis of total phenolics, flavonoids content and antioxidant capacity

#### 2.7.1 Determination of total phenolics and flavonoids

Total phenolics (TPC) and flavonoids content (TFC) of TCH samples were assessed following the protocol previously published by Cucu et al. (47). A calibration curve was constructed using various concentrations (0.01–0.25 mg/mL) of gallic acid (y = 10.567x + 0.1268, R2 = 0.998) and rutin (y = 9.0645x + 0.0816, R2 = 0.999). The results were presented as gallic acid equivalents (GAE) in mg/100 g of honey for phenolics determination and rutin equivalents (RE) in mg/100 g of honey for flavonoids measurement.

#### 2.7.2 Determination of antioxidant capacity

DPPH/ABTS+ reflect radical scavenging in hydrophilic systems, while FRAP assesses reduction potential—collectively covering honey’s antioxidant mechanisms (12). The antioxidant capacity of TCH samples was determined by FRAP, DPPH, and ABTS+ methods. For the DPPH experiment, the procedure followed the method described by Hu et al. (48). The ABTS+ and FRAP assays for TCH were assessed utilizing commercial assay kits. The results were displayed as 50% inhibitory concentration (IC50) of DPPH and ABTS+, while the FRAP results were expressed as mg/Trolox kg of honey.

### 2.8 Statistical analysis

Except for the moisture content analysis, all experiments were repeated with five biological replicates and the results are presented as mean ± standard error (SE). Data normality was confirmed via Shapiro–Wilk tests (*p* > 0.05). Unpaired t-test were performed using IBM SPSS (version 27, IBM Corp., NY, United States). Additionally, Pearson correlation coefficient was used for correlation analysis. The metabolomics analyses were conducted using the online platform MetaboAnalyst.^1^

The raw MS data were imported into Compound Discoverer 3.2 software (Thermo Fisher Scientific, CA, United States) for subsequent data calibration and data analysis. Firstly, the CD3.2 software identified and aligned the ion peaks in the raw file, as well as normalizing the peak area, and performed preliminary characterization. The ion peak matching parameters were as follows: maximum allowed retention time offset 0.2 min; maximum allowed mass deviation 5 ppm; minimum peak response value 1 × 10^7^; signal to noise ratio 3; peak response deviation 30%. The mzCloud, ChemSpider and mzVaul databases were used for metabolite identification.

A peak rating threshold of 4.5 was applied and only features detected in at least three samples were retained.

The qualitative volatiles were identified using the MassHunter Workstation Unknowns Analysis, B.09.00 software (Agilent Technologies, CA, United States) combined with the NIST 17 database, based on retention time, mass-spectral similarity match (> 80%) and Retention Index (RI) offsets of ± 20 units.

## 3 Results and discussion

### 3.1 Dewatering efficiency and energy consumption of honey cabinet

Following a 96-h treatment in the honey cabinet at a temperature of 38 °C ± 1 °C and relative humidity of 30% ± 5%, the water content of the capped honeycombs of TCH produced by Yongxiu and Anfu both decreased to below 18% (Table 1). It was significantly lower than the European Union standard of less than 20% (49). This reduction effectively prevented fermentation and degradation, thereby enhancing the quality and facilitating long period storage of TCH (50).

**Table 1:** Honey dewatering and total energy consumption for each test.

Location of TCH production	Treatment time (Water content, %)		Total energy consumed for 96 h (KW h)
	0 H (*n* = 30)	96 H (*n* = 30)	
Yongxiu, Jiujiang	21.79 ± 0.17^a^	17.72 ± 0.09^b^	12.56
Anfu, Ji’an	21.45 ± 0.09^a^	17.80 ± 0.10^b^	13.02

The same lowercase letter in the same line indicated no significant difference (*p*>0.05), different lowercase letters indicated significant difference (*p*<0.05).

The average energy consumption per run of cabinet was 12.8 kWh, translated to an operational cost of approximately $1.06 per run, based on the 2025 U. S. industrial electricity rates. It was a marked reduction compared to traditional hot rooms, which are estimated to be 25–35 kWh for comparable throughput (40). Each run could process approximately 50 kilograms of TCH, so the cabinet’s energy use was 0.26 kWh/kg and the 96 h treatment cost was $0.02 per kilogram, which is 48% lower than commercial vacuum drying units (0.5 kWh/kg) (17) and 85% lower than thermal heating machine ($0.135 per kilogram) (35).

While a duration of 96 h may seem long, this is a viable timeframe for high value honey batch production in small apiaries. Additionally, automation has minimized labor costs. Further optimization may include slightly higher temperatures and lower relative humidity (within safe limits to avoid HMF or glycerol) to potentially reduce processing time without compromising quality.

In summary, the honey cabinet not only guaranteed efficient dehydration of capped honeycombs but also maintained a comparatively low operational cost. It provided a practical solution to improve income for small-scale beekeepers facing TCH devaluation due to high water content. With a low purchase cost of only $ 210 and an easy one-touch operation, it is highly suitable for small apiaries and favorable for rapid and wide-scale dissemination in humid regions.

### 3.2 Palynological characterization and effects on physicochemical parameters of TCH

**Figure 2:**
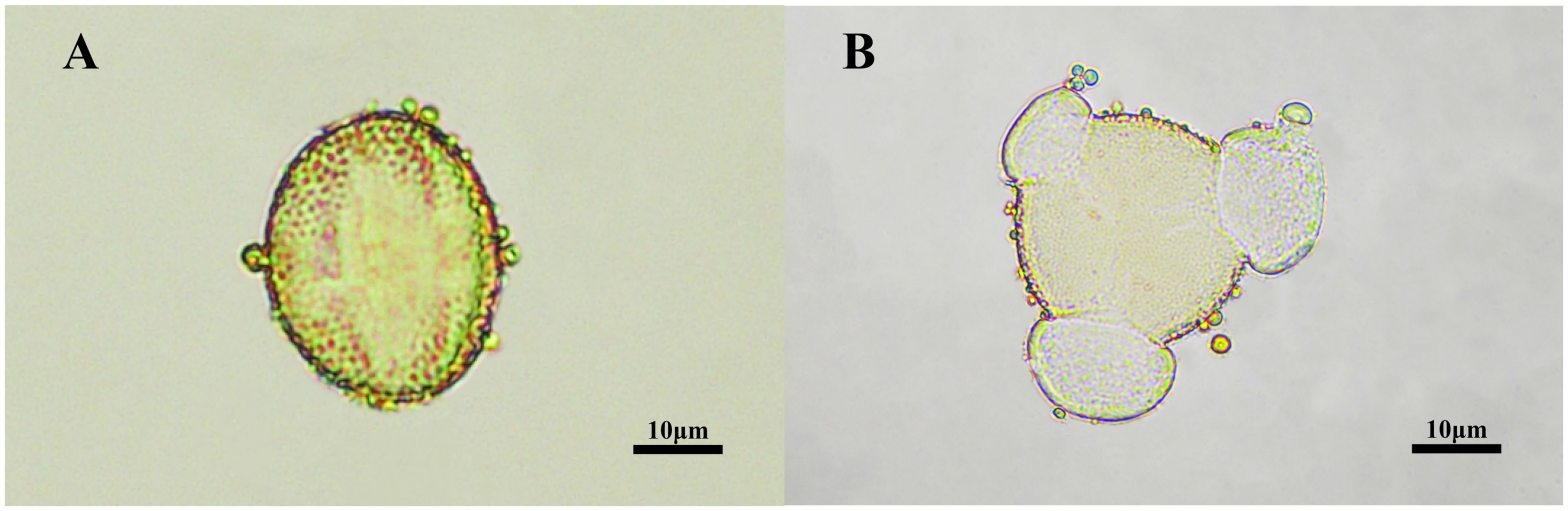
The palynological characterization of TCH. **(A)** An equatorial view of TCH pollen grains under the microscope. **(B)** A polar view of TCH pollen grains under the microscope.

The pollen grains in TCH observed under the microscope displayed a prolate shape in the equatorial view (Figure 2A) and a trilobed circular shape in the polar view (Figure 2B). The pollen grains of *T. cochinchinensis* accounted for 85.06% ± 2.51% in TCH. Which indicates that the samples met the requirement (>45%) to be considered monofloral honey (51).

Seven physicochemical parameters of TCH at different treatment times were shown in Table 2. As the treatment time increased, the content of fructose and glucose in TCH significantly increased, while the sucrose content significantly decreased. The sucrose was enzymatically hydrolyzed into fructose, glucose and other monosaccharide by invertase during the honey maturation process (52, 53). This indicated that during the honey cabinet treatment, TCH underwent a post maturation. Additionally, the significant increase in monosaccharide content aligned with the sugar composition characteristics observed in mature honey (54).

**Table 2:** Physicochemical parameters of 0 H and 96 H TCH.

Parameter		Treatment time	
		0 H (*n* = 5)	96 H (*n* = 5)
Fructose (%)		37.19 ± 0.29^b^	38.44 ± 0.15^a^
Glucose (%)		35.83 ± 0.37^b^	37.69 ± 0.21^a^
Sucrose (%)		0.73 ± 0.02^a^	0.49 ± 0.07^b^
HMF (5-hydroxymethylfurfural) (mg/kg)		ND	ND
Diastase activity mL/(g·h)		0.86 ± 0.17^b^	1.02 ± 0.18^a^
Free acidity (mL/kg)		15.19 ± 0.57^b^	18.07 ± 0.88^a^
Glycerol (mg/kg)		61.68 ± 1.05^a^	66.43 ± 2.60^a^
Color	L* (lightness)	95.35 ± 0.10^a^	95.34 ± 0.11^a^
	a* (Redness)	−2.15 ± 0.04^a^	−2.16 ± 0.01^a^
	b* (yellowness)	19.39 ± 0.21^b^	21.06 ± 0.09^a^

ND indicates data not detected. The same lowercase letter in the same line indicated no significant difference (*p*>0.05), different lowercase letters indicated significant difference (*p*<0.05).

HMF, a toxic cyclic aldehyde and an intermediary product of Maillard reactions in honey, served as a crucial indicator for evaluating the extent of honey thermal treatment (55, 56). No HMF was detected in any TCH samples, indicating that the honey cabinet treatment did not result in thermal degradation and consequently prevented the formation of harmful substances such as HMF (57).

Diastase is sensitive to heat, and exposure to thermal treatment can cause denaturation of its structure, leading to inactivation (15, 58). The activity of diastase is frequently used as an indicator to evaluate the freshness and the processing suitability of honey (59). TCH naturally exhibits low diastase activity (18). As treatment time increased, the diastase activity in TCH also rose, which is consistent with Zhang et al.’s findings that diastase activity increases with greater maturity (60).

Acidity plays a pivotal role in inhibiting microbial growth (51). The significant increase in acidity of 96 H TCH enhanced the antibacterial properties of honey (61). Honey fermentation results in the production of glycerol, which is difficult to eliminate during subsequent honey processing (62). It can be used to assess honey spoilage and processing suitability (63). The glycerol content of TCH showed no difference after 96 h of processing, which indicates that it will not accelerate fermentation and extend the storage time. The color of TCH is light amber (64). The b* (yellowness) values increased significantly with prolonged treatment time, suggesting that TCH is progressively maturing.

### 3.3 Variation of volatile compounds of TCH

Volatile compounds impart both taste and aroma to honey, representing essential quality characteristics of honey (65). Over 600 volatile compounds have been identified in various types of honey (66, 67).

A total of 8 kinds of 36 volatile compounds were tentatively identified from 0 H and 96 H TCH, including acids, alcohols, aldehydes, esters, hydrocarbons, aromatics, phenols and ethers. All of them were present on both time points. Both groups identified octanal and nonanal as frequently detected volatile compounds, contributing to the fresh citrus-like fruit aroma characteristic of TCH (46, 68). Furthermore, nonanoic acid and decanol impart woody and floral aromas to TCH (69, 70).

**Figure 3:**
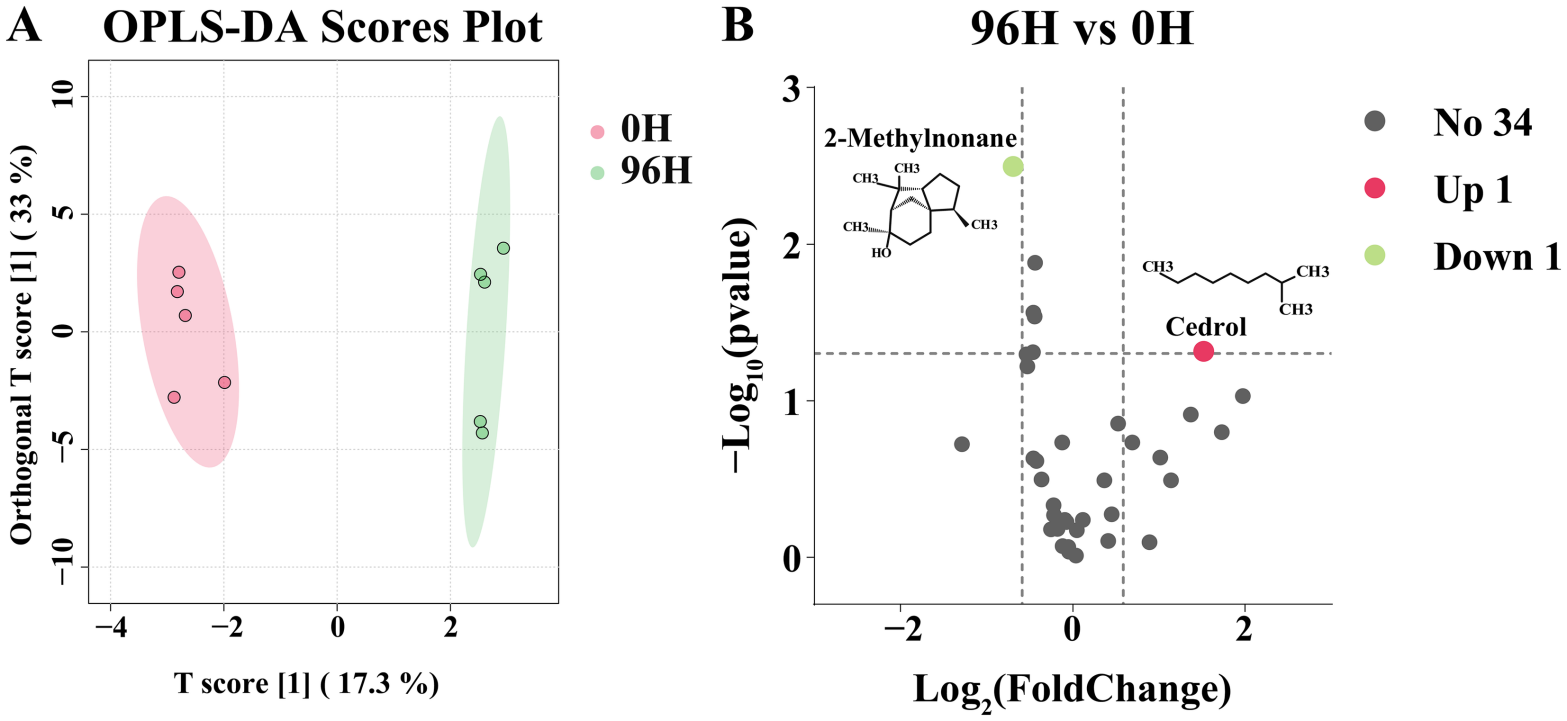
The GC–MS analysis of 0 H and 96 H TCH. **(A)** The OPLS-DA scores plot of volatile compounds determined by GC–MS of 0 H and 96 H TCH (*n* = 5). **(B)** Volcano plot of 36 identified volatile compounds from 96 H TCH vs. 0 H TCH. The differential volatile compounds were analyzed by *p* < 0.05 and |log2FC| > 0.585.

The orthogonal partial least squares-discriminant analysis (OPLS-DA) was carried out on 36 volatile compounds. The two groups were clearly separated in the OPLS-DA score plot, indicating the discrimination between them was excellent (Figure 3A). The differential volatile compounds were screened using *p* < 0.05 and |log2FC| > 0.585. Compared to the 0 H TCH, the 96 H TCH had 2 differential volatile compounds (Figure 3B). The proportion of non-differential volatile compounds was 94.44%. 2-methylnonane was down-regulated. Hydrocarbons are common in honey and may originate from flower nectar and further converted by bees (71). The result was consistent with the studies that the maturity of Gallnut honey increased while hydrocarbons content decreased (72). The content of cedrol increased after 96 H treatment. Previous studies have shown that the volatile compounds in honey, particularly terpenoids, alcohols, and aldehydes, fluctuate during the ripening process, consistent with our findings (73). Cedrol provides herbal flavors for TCH and serves as a volatile marker and an important contributor to unifloral safflower honey (74). Therefore, it was observed that after 96 H treatment by honey cabinet, the overall volatile compounds of TCH had no significant alterations.

Sensory evaluation was valuable for flavor perception. However, the minimal changes in key aroma-active compounds in TCH like octanal, nonanal and nonanoic acid (65), along with the increase in cedrol, indicate that the overall sensory profile characteristic of TCH is well-preserved (75). Moreover, sensory perception depends on both compound concentrations and odor thresholds, and given that 2-methylnonane is a minor component, its sensory impact is likely to be limited (76). Future studies should include sensory panels to confirm consumer acceptability.

### 3.4 Variation of chemical compositions of TCH

**Figure 4:**
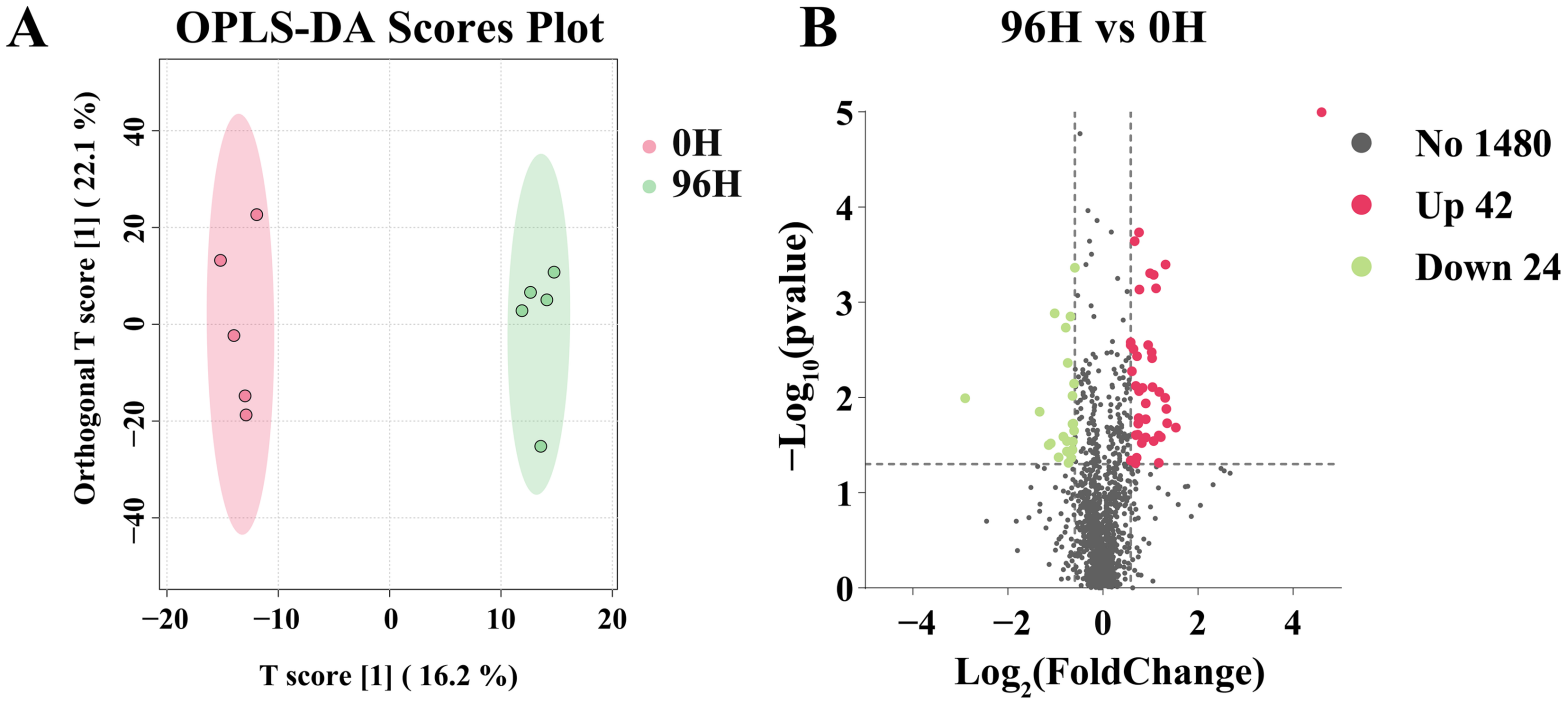
The UPLC-MS/MS analysis of 0 H and 96 H TCH. **(A)** The OPLS-DA scores plot of compounds determined by UPLC- MS/MS of 0 H and 96 H TCH (*n* = 5). **(B)** Volcano plot of the 1,546 identified metabolites from 96 H TCH vs. 0 H TCH. The differential metabolites were analyzed by *p* < 0.05 and |log2FC| > 0.585.

A total of 1,546 small molecule compounds were identified in TCHs (0 H and 96 H treatment), with the composition of components being identical in both groups. The OPLS-DA analysis was carried out on the 1,546 metabolites in two groups. There was a clear separation in the OPLS-DA score plot, indicating that the optimal classification result has been achieved (Figure 4A). The differential metabolites were screened using *p* < 0.05 and |log2FC| > 0.585. Compared to the 0 H TCH, the 96 H TCH exhibited 66 differential components (Supplementary Table S2; Figure 4B), including 42 up-regulated components, mainly organic acids, fatty acids, esters, phenolic acids, flavonoids and others. The proportion of non-differential compounds was 95.73%.

The upregulation of differential components in the 96 H TCH could primarily be attributed to two factors. Firstly, the reduction in water content resulted in a higher concentration of specific components. Secondly, after 96 h of treatment, TCH had exhibited greater transformation and maturity. For example, during the maturation process, sucrose continuously undergoes hydrolysis to form fructose and glucose, while organic acids are simultaneously generated (77). The elevated levels of six organic acids contributed to lowering the pH value of TCH, thereby enhancing its antibacterial properties (78). Fatty acids accumulate by the action of lipase on lipids during honey maturation, and the subsequent dehydration process further increases their concentration (79). There was a significant upregulation of two long-chain fatty acids, which aligned with previous research that mature honey contained significantly higher levels of long-chain fatty acids compared to immature honey (54). Additionally, the levels of three esters in the 96 H TCH had also increased, contributing to the development of TCH’s distinctive fruity and floral aromas (80). Furthermore, we also found that one Phenolic acid and one flavonoid were upregulated in the 96 H TCH, consistent with the finding that some polyphenol levels increase during TCH maturation (81), potentially improving the antioxidant capacity of TCH. Phenolic increases may arise from continued plant enzyme activity (e.g., polyphenol oxidase) during dehydration, converting glycosides to aglycones (82).

These results collectively suggested that honey cabinet treatment promoted honey maturation without causing significant changes in the composition or content of the compound and may enhance TCH antioxidant capacity.

### 3.5 Comparative analysis of TPC, TFC and antioxidant capacity of TCH

**Figure 5:**
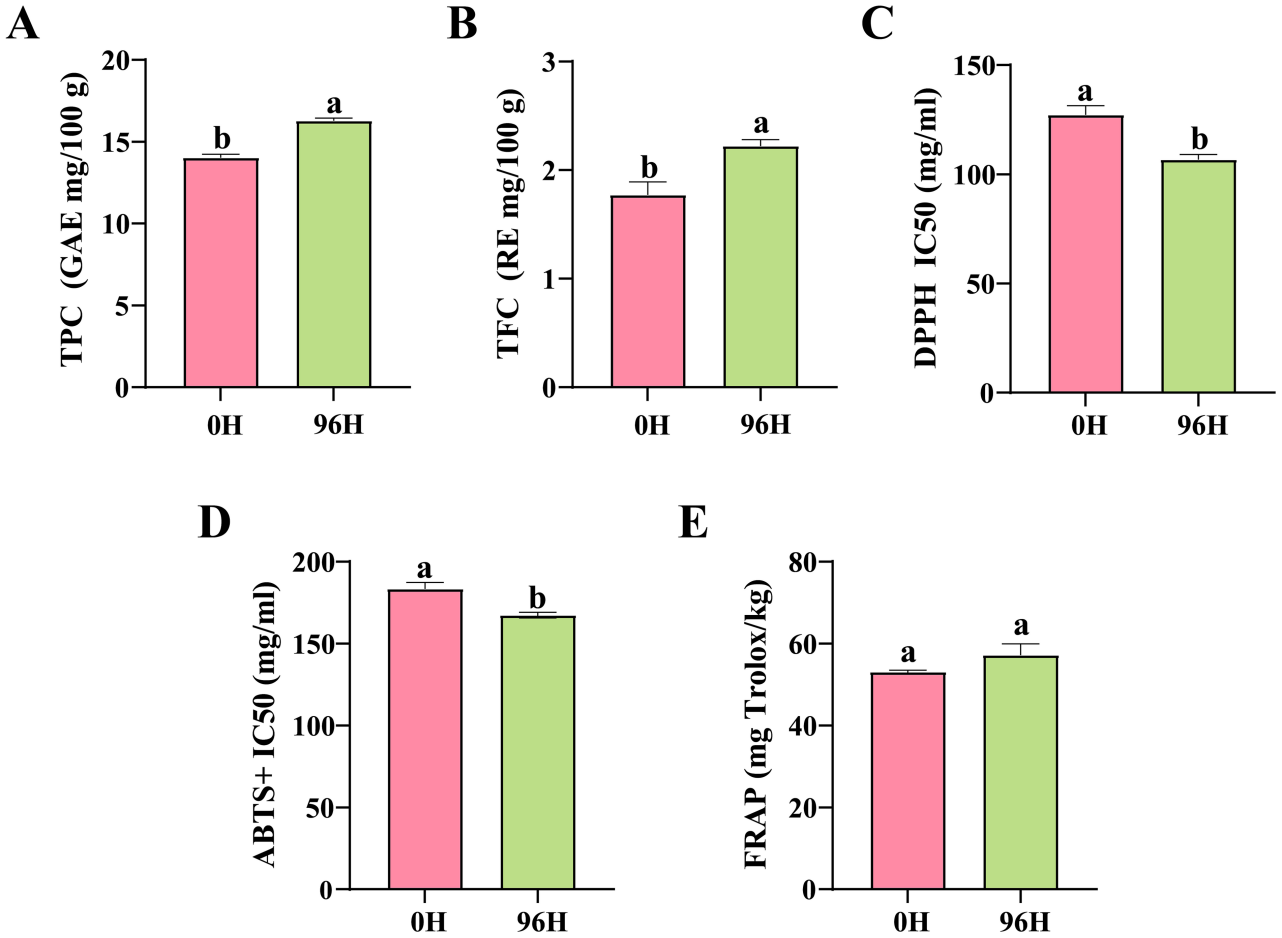
Total phenolics, flavonoids content and antioxidant capacity of 0 H and 96 H TCH. **(A)** Total phenolics content (GAE mg/100 g) in 0 H and 96 H TCH (unpaired t-test, *n* = 5). **(B)** Total flavonoids content (RE mg/100 g) in 0 H and 96 H TCH (unpaired t-test, *n* = 5). **(C)** The 2,2-diphenyl-1-picrylhydrazyl (DPPH) IC50 (mg/mL) in 0 H and 96 H TCH (unpaired t-test, *n* = 5). **(D)** The ABTS free radical scavenging capacity (ABTS+) IC50 (mg/mL) in 0 H and 96 H TCH (unpaired t-test, *n* = 5). **(E)** The ferricion reducing antioxidant power (FRAP; mg Trolox/kg) in 0 H and 96 H TCH (unpaired *t*-test, *n* = 5). The same lowercase letter in the same figure indicated no significant difference (*P*>0.05), different lowercase letters indicated significant difference (*P*<0.05).

Phenolic acids and flavonoids are important bioactive substances in honey, mainly contributing to its antioxidant activity (83, 84). The TPC and TFC of the 96 H TCH were significantly increased by 15.83 and 25.42%, respectively, compared to those of the 0 H TCH (Figures 5A,B). Based on the UPLC- MS/MS results, methyl gallate and 2-methoxyisoliquiritigenin showed significant increase. Furthermore, phenolic acids like gallic acid, sinapinic acid and 4-hydroxy-2-methylbenzoic acid, as well as flavonoids including quercetin, kaempferol, eupatorin and sakuranetin, all exhibited upward trends. Methyl gallate (upregulated 2.1-fold) exhibits high oral bioavailability and antimicrobial effects against *Aspergillus* spp., potentially extending shelf-life (85).

The DPPH IC50 and ABTS+ IC50 values for 96 H TCH are notably lower by 19.20 and 9.5%, respectively, compared to those of 0 H TCH (Figures 5C,D). Although the FRAP values did not reach statistical significance, they exhibited an upward trend (Figure 5E). As shown in Table 3, both TPC and TFC in TCH have significant correlations with antioxidant capacity (DPPH IC50, ABTS+ IC50 and FRAP). These results showed that 96 h treatment with honey cabinet enhanced not only the TPC and TFC, but also the *in vitro* antioxidant capacity.

**Table 3:** Correlation matrix between TPC, TFC, and antioxidant capacity in TCH (*n* = 5).

Parameter	TPC	TFC	DPPH IC50	ABTS^+^ IC50	FRAP
TPC	1				
TFC	0.924**	1			
DPPH IC50	−0.945**	−0.868*	1		
ABTS+ IC50	−0.965**	−0.837*	0.883*	1	
FRAP	0.946**	0.980**	−0.906*	−0.858*	1

Level of significance: *p* < 0.05 (2-tailed): Groups marked with a single asterisk (*); *p* < 0.01 (2-tailed): Groups marked with a double asterisk (**).

Two factors may explain this observation: firstly, the treatment may improve honey maturity. Guo et al. noted that mature honey possessed richer polyphenolic compositions, and Zhang et al. found the TPC, TFC and vitro antioxidant capacity of rape honey displayed an overall upward trend with the increase of ripening (60, 82). Secondly, the b* (yellowness) values of 96 H TCH significantly increased. Research indicates that the color values of honey is a positive correlation with phenolic compounds content and antioxidant capacity (86). Estevinho et al. demonstrated that dark honeys contain significantly higher phenolics and superior DPPH scavenging capacity than light variants (87).

## 4 Conclusion

In this study, after 96 h of treatment at the temperature of 38 ± 1 °C and relative humidity of 30 ± 5% in the honey cabinet, the water content of TCH decreased to below 18%, effectively extending its shelf life. Results showed that this dewatering method not only accelerated TCH maturation and improved quality but also avoided the adverse effects of conventional thermal treatment. The composition and content of chemical composition and volatile compounds remained largely unchanged, with 95.73 and 94.44% similarity before and after treatment, respectively. Additionally, it increased TPC and TFC, enhancing antioxidant activity. These findings confirmed the honey cabinet’s effectiveness and practicality for TCH dewatering and quality preservation, providing a strong theoretical and technical foundation for its broader application in small apiaries. Additionally, it shows great potential in handling other high water honeys across Southeast Asia. While the sample size (30 honeycombs) and biological replication (*n* = 5) provide statistically significant results for the parameters measured, future studies with larger-scale validation across multiple seasons would strengthen generalizability.

Beekeepers can adopt this system for $210 (material costs), recovering investments within one season via improved honey quality. To ensure optimal performance, it is recommended that beekeepers follow the specified temperature and humidity settings when operating the honey cabinet. During the dewatering treatment, beekeepers should regularly check the water content of honeycombs to avoid over-drying. Overall, the cabinet’s cost-effectiveness and scalability provide a viable solution for small apiaries, enhancing economic returns for beekeepers in humid regions.

## Data Availability

The original contributions presented in the study are included in the article/Supplementary material, further inquiries can be directed to the corresponding authors.

## Footnotes

^1^ https://www.metaboanalyst.ca/

## Supplementary material

SUPPLEMENTARY FIGURE S1 The structural diagram of honey cabinet includes left view, right view and air intake view. The meaning of numbers in the figure are as follows: 1. Dehumidifier, 2. Temperature and humidity sensor, 3. Axial fan, 4. Honeycomb shelves, 5. Heater, 6. Air intake, 7. Air outlet.

SUPPLEMENTARY FIGURE S2 The 3D schematic representations: left and right view of honey cabinet, and control components including heaters, dehumidifiers, axial fans, air intake and air outlet.

SUPPLEMENTARY FIGURE S3 The distribution of relevant parameters within honey cabinet. **(A)** The airflow mechanism of honey cabinet. Air enters through the bottom intake, accelerated by an axial fan to form a horizontal laminar flow that evenly spreads across the base and gradually fills the cabinet vertically, finally being discharged directionally through diagonally positioned upper vents. **(B)** The distribution of temperature in honey cabinet (°C), (C) The distribution of humidity in honey cabinet (%).

SUPPLEMENTARY FIGURE S4 The internal temperature and relative humidity within the honey cabinet during a 96-h operation (38 °C ± 1 °C and the RH 30 ± 5%).

SUPPLEMENTARY FIGURE S5 The operational flow diagram for the honey cabinet.
