# Supplementary material for: Enhancing antioxidant activity and quality of Triadica cochinchinensis honey via an automated temperature-humidity controlled cabinet
Source: Front Nutr. 2025 Sep 24;12:1641551. doi: 10.3389/fnut.2025.1641551 (PMC12507334; doi:10.3389/fnut.2025.1641551)
Supplement: SUPPLEMENTARY FIGURE S1 — The structural diagram of honey cabinet includes left view, right view and air intake view. The meaning of numbers in the figure are as follows: 1. Dehumidifier, 2. Temperature and humidity sensor, 3. Axial fan, 4. Honeycomb shelves, 5. Heater, 6. Air intake, 7. Air outlet. [file Data_Sheet_1.zip › Supplementary Figures and Tables-R1/Table S1.docx]

**Table S1**. Relevant parameters of honey cabinet.

| **Technical Parameters** | **Unit** | **Value** |
| --- | --- | --- |
| External dimensions (L × W × H) | mm^3^ | 600×800×1350 |
| Temperature control range and accuracy | ℃ | 38 (±1) |
| Relative humidity control range and accuracy | % | 30 (±5) |
| Internal hot air wind speed | m/s | 0.6-1.2 |
| Drying chamber volume | m^3^ | 0.46 |
| Total power | w | 500 |
