# Supplementary material for: Enhancing antioxidant activity and quality of Triadica cochinchinensis honey via an automated temperature-humidity controlled cabinet
Source: Front Nutr. 2025 Sep 24;12:1641551. doi: 10.3389/fnut.2025.1641551 (PMC12507334; doi:10.3389/fnut.2025.1641551)
Supplement: SUPPLEMENTARY FIGURE S1 — The structural diagram of honey cabinet includes left view, right view and air intake view. The meaning of numbers in the figure are as follows: 1. Dehumidifier, 2. Temperature and humidity sensor, 3. Axial fan, 4. Honeycomb shelves, 5. Heater, 6. Air intake, 7. Air outlet. [file Data_Sheet_1.zip › Supplementary Figures and Tables-R1/Table S2.docx]

**Table S2:** Information for 66 differential metabolites in 0 H and 96 H TCH identified by UPLC-MS/MS.

| **Number** | **Name** | **Formula** | **FC** | **P-value** |
| --- | --- | --- | --- | --- |
| 1 | Imidazo[1,2-a] pyrazine | C6H5N3 | 24.390 | 1.00E-05 |
| 2 | 1-[2-(2,5-Dimethyl-1H-pyrrol-1-yl)-4-nitrophenyl]-4-(methylsulfonyl)piperazine | C17H22N4O4S | 2.907 | 2.06E-02 |
| 3 | Diethylene glycol | C4H10O3 | 2.551 | 1.86E-02 |
| 4 | (4-Benzyl-1-piperidinyl) [1-(benzylsulfonyl)-3-piperidinyl] methanone | C25H32N2O3S | 2.525 | 1.32E-02 |
| 5 | 7-Hydroxy-6-methoxy-2-oxo-2H-chromene-3-carboxylic acid | C11H8O6 | 2.500 | 4.00E-04 |
| 6 | (5S,6S)-5-hydroxy-4-methoxy-6-(2-phenylethyl)-5,6-dihydro-2H-pyran-2-one | C14H16O4 | 2.481 | 1.00E-02 |
| 7 | N-(cyclopropylmethyl)-N'-phenylurea | C11H14N2O | 2.326 | 2.60E-02 |
| 8 | 6-(Ethoxycarbonyl)-3-hydroxy-4-oxo-4H-pyran-2-carboxylic acid | C9H8O7 | 2.278 | 8.72E-03 |
| 9 | 4-[5-Hydroxy-6-(2-hydroxy-3-methyl-4-methylenetetrahydro-2-furanyl)-1,3-dioxan-4-yl]-2,6-piperidinedione | C15H21NO7 | 2.268 | 2.50E-02 |
| 10 | 4-methoxyphenyl 3,5-dimethyl-1-phenyl-1H-pyrazole-4-carboxylate | C19H18N2O3 | 2.257 | 4.86E-02 |
| 11 | ethyl 2-(tert-butyl)-3-chloro-5-cyano-6-morpholinoisonicotinate | C17H22ClN3O3 | 2.174 | 7.09E-04 |
| 12 | perlolyrine | C16H12N2O2 | 2.105 | 2.86E-02 |
| 13 | N-(4-chlorophenethyl)-N'-(4-chlorophenyl) urea | C15H14Cl2N2O | 2.096 | 5.12E-04 |
| 14 | N4-(2-Methoxyphenyl)-7-nitro-2,1,3-benzoxadiazol-4-amine | C13H10N4O4 | 2.066 | 7.72E-03 |
| 15 | 1,2,4-Benzenetricarboxylic acid | C9H6O6 | 2.049 | 3.83E-03 |
| 16 | Methyl(1a′S,2R,3S,5′S,6a′S)-5′-(β-D-glucopyranosyloxy)-3-hydroxy-1a′,5′,5a′,6a′-tetrahydro-1b′H-spiro [oxirane-2,6′-oxireno [3,4] cyclopenta[1,2-c] pyran]-2′-carboxylate | C17H22O12 | 2.037 | 3.34E-03 |
| 17 | 3-(2-(Phenylamino) oxazol-5-yl) benzonitrile | C16H11N3O | 1.992 | 4.93E-04 |
| 18 | 5-acetyl-2,6-dimethyl-1,2,3,4-tetrahydropyridin-4-one | C9H13NO2 | 1.934 | 2.80E-03 |
| 19 | 4-oxododecanedioic acid | C12H20O5 | 1.873 | 1.15E-02 |
| 20 | 2-amino-N-(1,3,5-trimethyl-1H-pyrazol-4-yl) benzamide | C13H16N4O | 1.866 | 2.61E-02 |
| 21 | 4'-Hydroxy-2'-methoxychalcone | C16H14O3 | 1.866 | 1.68E-02 |
| 22 | 2-Furanbutanoic acid, a,g-dioxo- | C8H6O5 | 1.786 | 7.89E-03 |
| 23 | 2-Deoxy-2-({N-[2-(5,5-dimethyl-1,3-dioxan-2-yl) ethyl]-L-alanyl} amino)-D-glucopyranose | C17H32N2O8 | 1.764 | 3.00E-02 |
| 24 | (6S)-6-[(1S,2R)-1,2-dihydroxypentyl]-4-methoxy-5,6-dihydro-2H-pyran-2-one | C11H18O5 | 1.751 | 2.92E-02 |
| 25 | 9,10-Dihydroxystearic acid | C18H36O4 | 1.709 | 7.31E-04 |
| 26 | 10,16-Dihydroxyhexadecanoic acid | C16H32O4 | 1.695 | 1.83E-04 |
| 27 | Diphenol glucuronide | C12H14O8 | 1.692 | 8.50E-03 |
| 28 | Gynocardin | C12H17NO8 | 1.692 | 8.50E-03 |
| 29 | 3-(1,1,1-Trifluoro-2-propanyl) dihydro-2(3H)-furanone | C7H9F3O2 | 1.684 | 1.64E-02 |
| 30 | 3-Methyl-N-{[(2-methyl-2-propanyl) oxy] carbonyl}-L-valyl-(4R)-N-(1R,2S)-1-[(cyclopropylsulfonyl)carbamoyl]-2-vinylcyclopropyl-4-hydroxy-4-(4-phenoxyphenyl)-L-prolinamide | C37H48N4O9S | 1.681 | 1.87E-02 |
| 31 | 2-(2-{2-[2-(2-methoxy-ethoxy)-ethoxy]-ethoxy -ethoxy)-ethano | C11H24O6 | 1.669 | 2.46E-02 |
| 32 | 8-Isoprostaglandin F1β | C20H36O5 | 1.650 | 3.68E-03 |
| 33 | Radicinin | C12H12O5 | 1.639 | 4.29E-02 |
| 34 | Methyl(2R)-2-methyl-4-[(1S,5R,6R)-1,5,6-trihydroxy-4-methyl-2-oxo-3-cyclohexen-1-yl] butanoate | C13H20O6 | 1.623 | 7.53E-03 |
| **Number** | **Name** | **Formula** | **FC** | **P-value** |
| 35 | N-({(2R,3S,4R,5S)-3,4-Dihydroxy-5-[2-(isopropylamino)-2-oxoethyl] tetrahydro-2-furanyl} methyl) cyclopentanecarboxamide | C16H28N2O5 | 1.621 | 2.47E-02 |
| 36 | 2-[(4,5-dimethyl-2-furyl) methylidene]-5,5-dimethylcyclohexane-1,3-dione | C15H18O3 | 1.608 | 4.88E-02 |
| 37 | (Trifluoroacetyl)acetone | C5H5F3O2 | 1.587 | 2.28E-04 |
| 38 | Methyl gallate | C8H8O5 | 1.565 | 3.08E-03 |
| 39 | (3R,15R)-3,15-dihydroxypalmitic acid | C16H32O4 | 1.529 | 5.29E-03 |
| 40 | 3,4-Bis(4-propan-2-ylanilino) cyclobut-3-ene-1,2-dione | C22H24N2O2 | 1.506 | 4.58E-02 |
| 41 | 2-Hexyl-1,2,3,4-tetrahydronaphthalene | C16H24 | 1.506 | 2.62E-03 |
| 42 | N-(1-[N-benzyl-N-(1,3-thiazol-5-ylmethyl) glycyl]-5-(2-methylphenyl)-1,2,3,6-tetrahydropyridin-4-yl} carbonyl)-L-methionine | C31H36N4O4S2 | 1.502 | 2.79E-03 |
| 43 | Methyl[3R,6aS,8R,10aS)-3-hydroxy-1-(3,3,3-trifluoropropyl) decahydropyrano[2,3-c] [1,5] oxazocin-8-yl] acetate | C15H24F3NO5 | 0.664 | 4.33E-04 |
| 44 | Forchlorfenuron | C12H10ClN3O | 0.658 | 2.22E-02 |
| 45 | methyl 4,5-di(4-methoxyphenyl) thiophene-2-carboxylate | C20H18O4S | 0.656 | 7.09E-03 |
| 46 | 4-Coumaric acid | C9H8O3 | 0.651 | 1.87E-02 |
| 47 | Naringeninchalcone | C15H12O5 | 0.647 | 2.88E-02 |
| 48 | Coumarin | C9H6O2 | 0.644 | 1.89E-02 |
| 49 | Citroflex A-4 | C20H34O8 | 0.643 | 3.53E-02 |
| 50 | (±)9-HpODE | C18H32O4 | 0.641 | 9.60E-03 |
| 51 | (E)-Stilbestrol | C14H12O2 | 0.633 | 4.30E-02 |
| 52 | 3,5-Dihydroxybenzoic acid | C7H6O4 | 0.623 | 1.40E-03 |
| 53 | (2-Dodecen-1-yl) succinic anhydride | C16H26O3 | 0.612 | 3.78E-02 |
| 54 | tetranor-12(R)-HETE | C16H26O3 | 0.612 | 3.78E-02 |
| 55 | 2,6-Di-tert-butyl-1,4-benzoquinone | C14H20O2 | 0.607 | 4.87E-02 |
| 56 | (2E)-decenoic acid | C10H18O2 | 0.600 | 4.31E-03 |
| 57 | 5-(hydroxymethyl)-4-methoxy-2,5-dihydrofuran-2-one | C6H8O4 | 0.592 | 3.68E-02 |
| 58 | 3,4-Dihydrocadalene | C15H20 | 0.592 | 2.85E-02 |
| 59 | 3-(3,4-dihydroxyphenyl) propanoic acid | C9H10O4 | 0.581 | 1.83E-03 |
| 60 | Benzyl formate | C8H8O2 | 0.561 | 2.57E-02 |
| 61 | Cholestan-3-ol | C27H48O | 0.525 | 4.24E-02 |
| 62 | Indene | C9H8 | 0.497 | 1.30E-03 |
| 63 | cyclohexylbenzen | C12H16 | 0.469 | 3.02E-02 |
| 64 | 10-HDA | C10H18O3 | 0.454 | 3.14E-02 |
| 65 | 5-Hydroxymethyl-2-furaldehyde | C6H6O3 | 0.398 | 1.40E-02 |
| 66 | (2E)-5-[8aS)-2,5,5,8a-tetramethyl-3-oxo-3,4,4a,5,6,7,8,8a-octahydronaphthalen-1-yl]-3-methylpent-2-enoic acid | C20H30O3 | 0.134 | 1.02E-02 |
