# Supplementary figures and images for: Enhancing antioxidant activity and quality of Triadica cochinchinensis honey via an automated temperature-humidity controlled cabinet
Source: Front Nutr. 2025 Sep 24;12:1641551. doi: 10.3389/fnut.2025.1641551 (PMC12507334; doi:10.3389/fnut.2025.1641551)

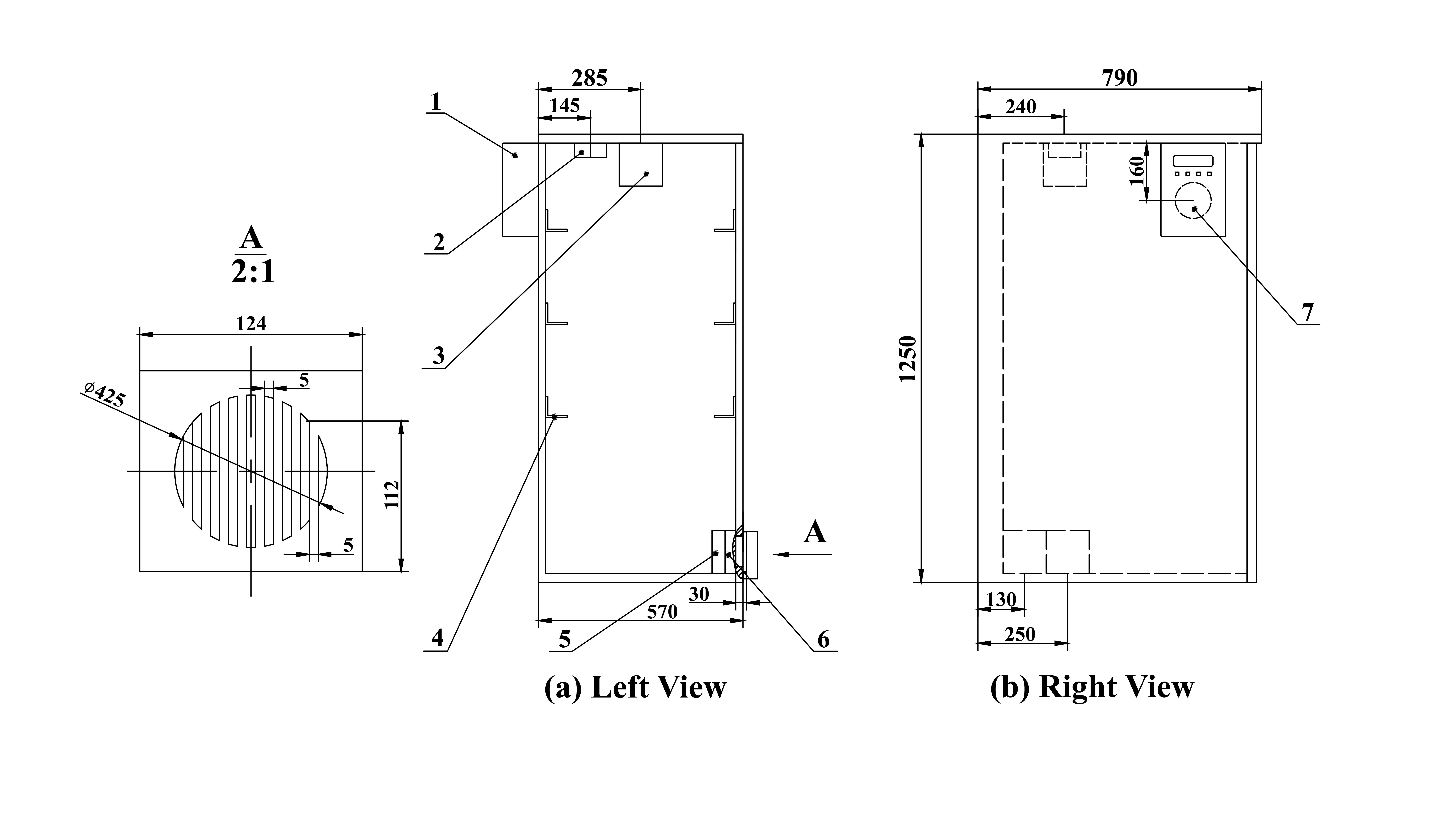

Supplement: SUPPLEMENTARY FIGURE S1 — The structural diagram of honey cabinet includes left view, right view and air intake view. The meaning of numbers in the figure are as follows: 1. Dehumidifier, 2. Temperature and humidity sensor, 3. Axial fan, 4. Honeycomb shelves, 5. Heater, 6. Air intake, 7. Air outlet. [file Data_Sheet_1.zip › Supplementary Figures and Tables-R1/Figure S1.jpg]

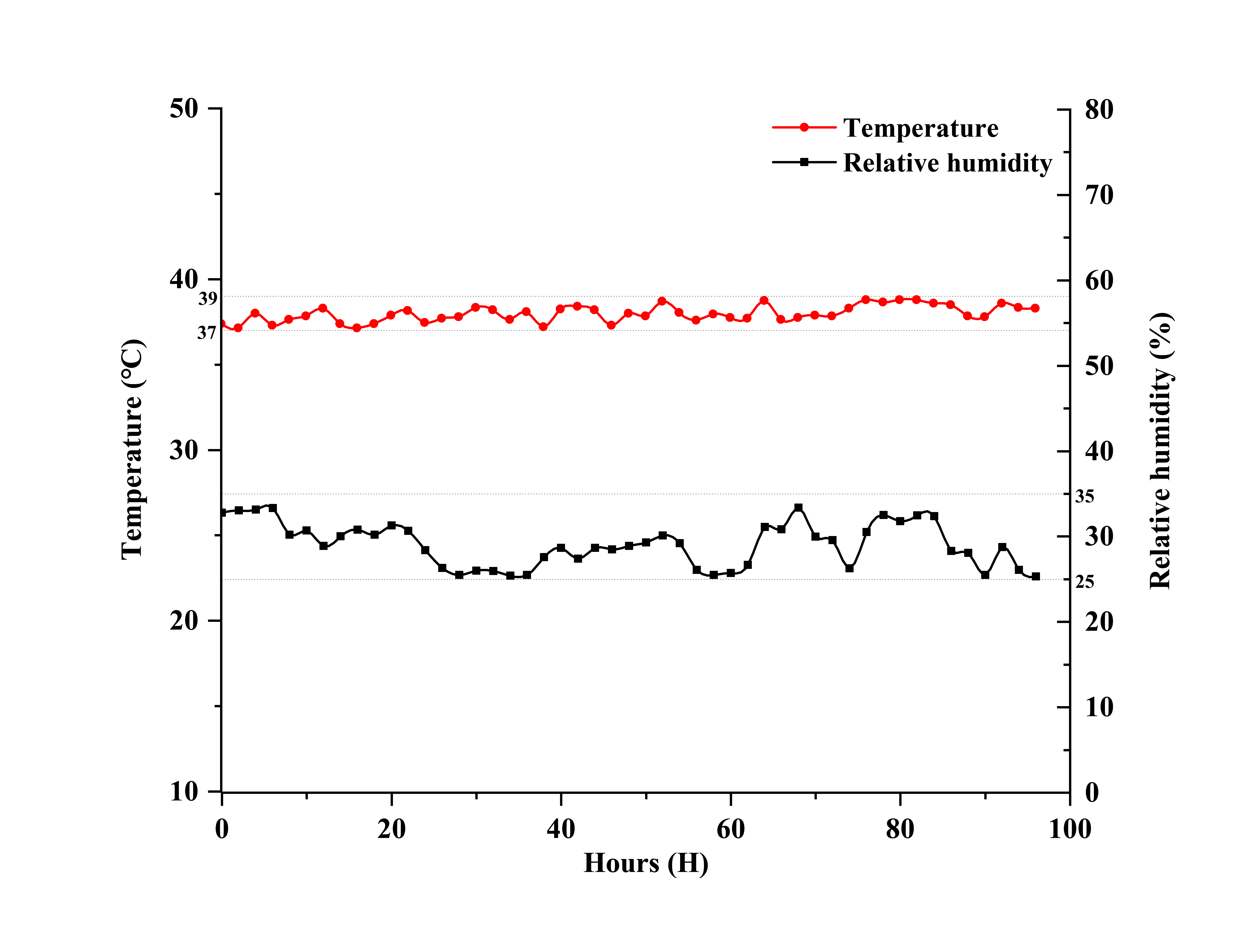

Supplement: SUPPLEMENTARY FIGURE S1 — The structural diagram of honey cabinet includes left view, right view and air intake view. The meaning of numbers in the figure are as follows: 1. Dehumidifier, 2. Temperature and humidity sensor, 3. Axial fan, 4. Honeycomb shelves, 5. Heater, 6. Air intake, 7. Air outlet. [file Data_Sheet_1.zip › Supplementary Figures and Tables-R1/Figure S4.jpg]

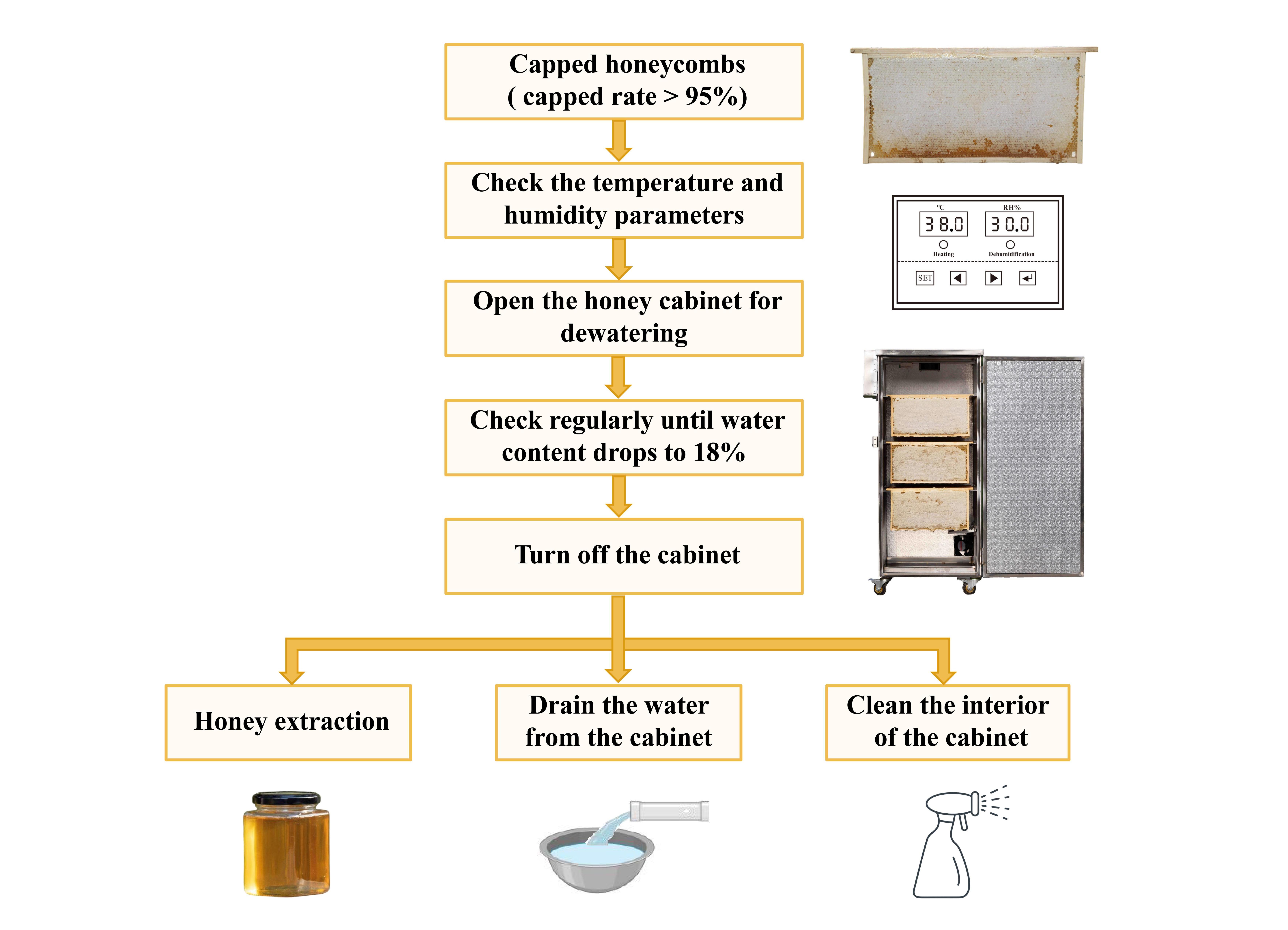

Supplement: SUPPLEMENTARY FIGURE S1 — The structural diagram of honey cabinet includes left view, right view and air intake view. The meaning of numbers in the figure are as follows: 1. Dehumidifier, 2. Temperature and humidity sensor, 3. Axial fan, 4. Honeycomb shelves, 5. Heater, 6. Air intake, 7. Air outlet. [file Data_Sheet_1.zip › Supplementary Figures and Tables-R1/Figure S5.jpg]
